# Effectiveness of AI-Driven Conversational Agents in Improving Mental Health Among Young People: Systematic Review and Meta-Analysis

**DOI:** 10.2196/69639

**Published:** 2025-05-14

**Authors:** Yi Feng, Yaming Hang, Wenzhi Wu, Xiaohang Song, Xiyao Xiao, Fangbai Dong, Zhihong Qiao

**Affiliations:** 1 Mental Health Center Central University of Finance and Economics Beijing China; 2 National Demonstration Center for Experimental Psychology Education (Beijing Normal University), Beijing Key Laboratory of Applied Experimental Psychology Faculty of Psychology Beijing Normal University Beijing China; 3 Lingxin AI Beijing China; 4 Mental Health Center Henan Agricultural University Henan China

**Keywords:** artificial intelligence, conversational agents, meta-analysis, mental health intervention, young people

## Abstract

**Background:**

The increasing prevalence of mental health issues among adolescents and young adults, coupled with barriers to accessing traditional therapy, has led to growing interest in artificial intelligence (AI)-driven conversational agents (CAs) as a novel digital mental health intervention. Despite accumulating evidence suggesting the effectiveness of AI-driven CAs for mental health, there is still limited evidence on their effectiveness for different mental health conditions in adolescents and young adults.

**Objective:**

This study aims to examine the effectiveness of AI-driven CAs for mental health among young people, and explore the potential moderators of efficacy.

**Methods:**

A total of 5 main databases (PubMed, PsycINFO, Embase, Cochrane Library, and Web of Science) were searched systematically dated from the establishment of the database to August 6, 2024. Randomized controlled trials comparing AI-driven CAs with any other type of control condition in improving depressive symptoms, generalized anxiety symptoms, stress, mental well-being, and positive and negative affect were considered eligible when they were conducted in young people aged 12-25 years. The quality of these studies was assessed using the Cochrane Risk of Bias tool. Data were extracted by 2 independent reviewers and checked by a third reviewer. Pooled effect sizes (Hedges *g*) were calculated using random effect models and visually presented in forest plots.

**Results:**

A total of 14 articles (including 15 trials) were included, involving 1974 participants. The results indicated that, after adjustment for publication bias, AI-driven CAs had a moderate-to-large (Hedges *g*=0.61, 95% CI 0.35-0.86) effect on depressive symptoms compared to control conditions. However, their effect sizes adjusting for publication bias for generalized anxiety symptoms (Hedges *g*=0.06, 95% CI –0.21 to 0.32), stress (Hedges *g*=0.002, 95% CI –0.19 to 0.20), positive affect (Hedges *g*=0.01, 95% CI –0.24 to 0.27), negative affect (Hedges *g*=0.07, 95% CI –0.13 to 0.27), and mental well-being (Hedges *g*=0.04, 95% CI –0.21 to 0.29) were all nonsignificant. Subgroup analyses revealed that AI-driven CAs were particularly effective in improving depressive symptoms among subclinical populations (Hedges *g*=0.74, 95% CI 0.50-0.98).

**Conclusions:**

The findings highlight the potential of AI-driven CAs for early intervention in depression among this population, and underscore the need for further improvements to enhance their efficacy across a broader range of mental health outcomes. Key limitations of the reviewed evidence include heterogeneity in therapeutic orientations of CAs and lack of follow-up measures. Future research should explore the long-term effects of AI-driven CAs on mental health outcomes.

## Introduction

Mental health issues among adolescents and young adults are increasingly becoming a public health concern, affecting between 10% and 20% of the global youth population [1]. The early-onset mental health disorders are particularly alarming, with 50% of cases emerging before the age of 14 years and 75% by the age of 25 years [2]. Despite the significant impact of mental health disorders on young populations, these conditions remain underdiagnosed and undertreated [3]. The impact of these untreated conditions is profound, as persistent mental health problems often extend into adulthood, leading to impairments in educational achievement, psychosocial functioning, and overall quality of life [4,5]. The COVID-19 pandemic has exacerbated these challenges, resulting in a marked increase in rates of depression, anxiety, and stress among young people [6].

In parallel with the rise in mental health issues, this generation of young people is growing up in a digital world. Over 90% of individuals aged 15-24 years are “online,” and even in low-income countries, mobile access is widespread [7,8]. Adolescents and young adults are also the earliest adopters and heaviest users of new technologies [9]. This level of digital engagement provides a unique opportunity to leverage digital mental health interventions, which can bridge the treatment gap by offering scalable, accessible, and cost-effective solutions [10]. Compared to traditional face-to-face therapy, web- and mobile-based interventions provide anonymity, reduce stigma, and offer greater flexibility and autonomy [11]. As a result, digital mental health interventions have gained increasing attention. For example, in the third global survey on eHealth, the World Health Organization (WHO) reported that 58% of the surveyed countries have integrated digital health strategies as part of their health care frameworks [12]. However, early forms of digital mental health interventions, such as internet-based cognitive behavioral therapy (CBT), encounter several challenges, including limited interactivity and relatively high dropout rates [13]. Furthermore, these interventions tend to be generalized, often lacking the personalization needed to meet the unique needs of individual users.

With the development of deep learning and natural language processing (NLP) techniques in the field of artificial intelligence (AI), a promising avenue for digital mental health interventions is the use of AI-driven conversational agents (CAs). These agents use AI to simulate human behavior and offer a task-oriented framework with evolving dialogue, enabling them to engage users in conversation [14]. These agents can provide psychoeducation and deliver treatment options [15], such as CBT. Based on the specific functionalities, operational modes, and application scenarios of “programs” (systems), current AI-driven CAs exhibit technical differentiation that mainly differs in system orientation and response pattern [16,17]. From the system-orientation perspective, AI-driven CAs divide into general purpose (eg, ChatGPT [OpenAI]) [18] and domain-specific types (eg, Woebot [Woebot Health]) [19], with the former possessing open dialogue capabilities and the latter focusing on specialized functional scenarios. Topic constraints manifest as globally open-type (eg, Replika [Luka, Inc]) [20] versus vertically constrained systems (eg, health care dialogue system MYLO companion site]) [21], where the latter uses keyword filtering and knowledge graphs to define topic boundaries. From the response-pattern perspective, AI-driven CAs encompass free dialogue architectures (eg, Elomia [Elomia Health, Inc]) [22] and structured-guided designs (eg, Mind Tutor) [23], with the latter implementing clinical intervention pathway control through dialogue trees and assessment scales. Training approaches may include supervised learning fine-tuning, reinforcement learning, and self-supervised learning. For instance, general-purpose AI-driven systems like ChatGPT use large-scale pretraining combined with human feedback reinforcement learning, while domain-specific systems like Tess [Pareto, inc] may integrate supervised learning with domain-specific data fine-tuning [24]. It should be specifically noted that these classification dimensions often demonstrate cross-integration in practical applications, making single-dimensional categorization challenging. For example, Woebot represents a domain-specific, topic-constrained, and structured-interaction CA that simultaneously incorporates CBT framework guidance and emotional support dialogue capabilities [19,25]. This study primarily focuses on whether the CA uses NLP technology. Compared with traditional non-NLP digital mental health interventions, NLP-enhanced systems exhibit superior contextual understanding of user inputs. These systems enable personalized, interactive support through therapeutic dialogues that emulate human communication patterns [26], demonstrating the potential to enhance therapeutic engagement and improve clinical outcomes [27]. Although these systems are increasingly used among adults, their effectiveness for adolescents and young adults remains underexplored. Given the increasing mental health burden and the unique digital engagement patterns of younger individuals, understanding the potential of AI-driven CAs to support mental health among this group is crucial.

Despite the growing interest in AI-driven CAs for mental health, there is still limited evidence on their effectiveness for various mental health conditions in adolescents and young adults. Previous reviews have often combined non-NLP and AI-driven CAs or included both young people and older adults [28,29], which may lead to significant heterogeneity. To address these gaps, this meta-analysis aims to evaluate the effectiveness of AI-driven CAs in reducing mental health symptoms, particularly depression and anxiety, among adolescents and young adults aged 12-25 years. In addition, this study explores the moderators that may influence treatment outcomes, such as characteristics of the study population and AI-driven CAs, to better understand the factors that enhance the intervention effectiveness of these digital tools among this population.

## Methods

### Literature Search

This study was not preregistered. This systematic review followed the PRISMA (Preferred Reporting Items for Systematic Reviews and Meta-Analyses) guidelines (Table S1 in Multimedia Appendix 1) [30]. To locate studies assessing the effectiveness of AI-driven CAs for mental health problems in adolescents and young adults, two independent researchers (LYN and SXH) conducted a comprehensive search across five databases: PubMed, PsycINFO, EMBASE, Cochrane Library, and Web of Science. The search spanned from the inception of each database up to August 6, 2024. The following search terms were used: (robot OR social bot OR dialogue system OR conversational agent OR conversational bot OR conversational system OR conversational interface OR chatbot OR chat bot OR chatterbot OR chatter bot OR chat-bot OR smartbot OR smart bot OR smart-bot OR virtual coach OR virtual agent OR embodied agent OR relational agent OR avatar OR virtual character OR animated character OR virtual human OR virtual assistant OR digital assistant OR counseling agent) AND (mental illness OR mental disorder OR affective disorder OR psychotic disorder OR posttraumatic stress disorder OR PTSD OR distress OR depress OR anxiety OR bipolar OR schizophrenia OR psychosis OR mental health OR mental wellness OR wellbeing OR well-being OR SWB OR happiness OR happy OR positive affect OR negative affect OR positive emotion OR negative emotion OR mood OR life satisfaction OR healthy relationship OR resilience OR self-efficacy). Detailed Search strategies were also provided in Multimedia Appendix 2. No filters were applied to ensure the inclusion of all relevant studies. In addition, reference lists of included studies and previous reviews were manually searched to identify any further eligible studies. A detailed search strategy is provided in the Multimedia Appendix 2.

### Inclusion and Exclusion Criteria

Studies were selected based on these criteria (Table 1):

1. Population: Studies using AI-driven CAs for managing mental health issues were included if the average age of participants was between 12 and 25 years. This age range followed previous meta-analyses conducted in adolescents and young adults [31]. No restrictions were imposed on the eligible participant populations regarding diagnoses of common mental disorders or any other clinical or demographic characteristics. Participants could be from clinical (formally diagnosed mental health conditions), subclinical (self-reported or screened mental health symptoms), or nonclinical populations.
2. Intervention: We included interventions delivered by AI-driven CAs. These CAs used AI technologies (eg, NLP or machine learning) in any way to direct the course of the agent’s conversation. Unlike traditional AI systems without NLP capabilities, these agents possess the capability to understand user intent, analyze contexts, and retrieve or generate appropriate responses based on the users’ input and the context of the conversation [28].
3. Comparator: Eligible studies included any control conditions, such as waitlist or active control groups (eg, treatment as usual, therapist-led interventions)
4. Outcomes: Studies were included if they reported at least one mental health outcome and provided sufficient data for effect size (ES) calculation.
5. Study design: Only randomized controlled trials (RCTs) were included. Studies on non-AI CAs, non-NLP CAs, review articles, conference abstracts, and non-English publications were excluded. We did not search for unpublished articles as their quality is relatively low and many of them are not peer-reviewed. The screening was performed independently by two researchers (WW and YH), and full texts of potential studies were obtained for detailed eligibility assessment.

**Table 1:** Inclusion and exclusion criteria based on the PICOS (Population, Intervention, Comparator, Outcome, and Study) framework and article type.

Criteria	Inclusion criteria	Exclusion criteria
Population	Average age of participants was 12-25 years	Average age was younger than 12 or older than 25 years
Intervention	AI^a^-driven CAs^b^ using NLP^c^ or ML^d^	Non-AI, Non-ML or Non-NLP CAs
Comparator	Any control conditions	No control condition
Outcomes	At least one mental health outcome with sufficient data was reported	No mental health outcome or insufficient data for ES^e^ calculation
Study design	Randomized controlled trials	Nonrandomized trials
Article type	Original research written in English	Unpublished articles

^a^AI: artificial intelligence.

^b^CA: conversational agent.

^c^NLP: natural language processing.

^d^ML: machine learning.

^e^ES: effect size.

### Data Extraction and Quality Assessment

For each included study, the following data were extracted: authorship, year of publication, participant characteristics (eg, sample size, gender distribution, and mean age), CA specifications (eg, name, platform, and interaction mode), intervention details (eg, length and control group type), and measures. Methodological quality was assessed using the Cochrane Risk of Bias tool [32], considering factors such as random sequence generation, allocation concealment, blinding of participants and assessors, handling of incomplete outcome data, and selective reporting. In total, 2 authors (WW and XS) independently conducted the data extraction and quality assessments. Disagreements were resolved through discussion, with the involvement of a third author (YH) when necessary. The results of the risk of bias assessments are visually presented in a summary graph.

### Meta-Analytic Procedure

For each study, the means, SDs, and sample sizes at posttest were extracted to compute ESs. As very few studies included a follow-up measurement and the follow-up intervals differed, follow-up ESs were not calculated. When multiple studies reported data for the same outcome, pooled ESs were calculated. Given the small sample sizes in some studies, Hedges *g* was used to adjust for bias [33]. All ESs were coded so that positive Hedges *g* values indicated superior outcomes for the treatment group relative to the control group. Where intention-to-treat and completer analyses were both available, data from the intention-to-treat analyses were used. Follow-up data were not analyzed due to insufficient reporting across studies. ES calculations and overall estimates were performed using Comprehensive Meta-Analysis software version 3.0 (Biostat, Inc) and Stata SE version 15.1 (StataCorp). For studies that did not report means and SDs, alternative statistics (eg, Cohen *d*, *t* values, *F* values) were used. Multiarm trials were treated in accordance with Cochrane guidelines by combining means and SDs to create a single pairwise comparison [34]. Given the expected heterogeneity across studies, a random-effects model was applied to estimate the mean ESs [35]. Heterogeneity was examined using the *Q* statistic and *I*² index [33]. Outliers were identified as studies whose 95% CIs lay outside the 95% CI of the overall estimate [36]. A “leave-one-out” sensitivity analysis was conducted to assess the robustness of the results. Subgroup and meta-regression analyses were performed with outliers excluded to explore possible causes of heterogeneity among studies.

To assess publication bias, three methods were used: (1) visual inspection of funnel plots; (2) Duval and Tweedie’s trim-and-fill procedure [37] to adjust ES estimates for publication bias; and (3) Egger test for funnel plot asymmetry [38]. Moderator analyses were performed for both categorical and continuous moderators where heterogeneity was significant (*P*<.10 or *I*²>25%) and the overall ES was significant (*P*<.05). Subgroup analyses used a mixed-effects model, while meta-regression used unrestricted maximum likelihood estimation. Moderators included age, gender, intervention length, interaction mode, delivery platform, sample type, and control group type, selected based on previous meta-analytic studies [28,39]. All visualization was conducted by R (RStudio, PBC) version 4.3.1 and Review Manager version 5.3 (The Cochrane Collaboration).

## Results

### Search Results

**Figure 1:**
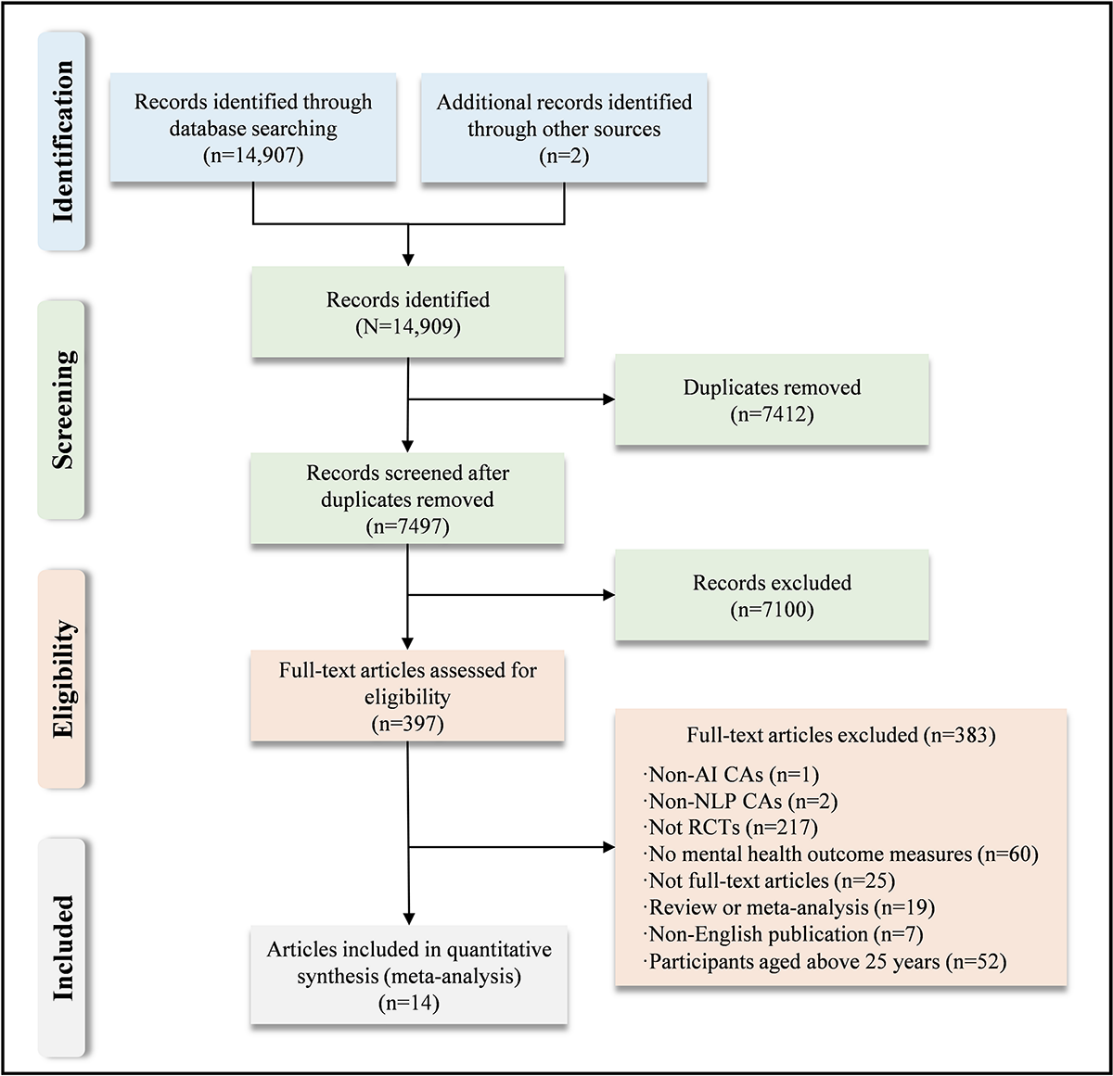
PRISMA (Preferred Reporting Items for Systematic Reviews and Meta-Analyses) flow diagram for the study. AI: artificial intelligence; CA: conversational agents; NLP: natural language processing; RCT: randomized controlled trials.

The initial database search identified 14,907 potentially relevant articles, supplemented by 2 additional studies found through manual reference list checks. The total sample size is 14,909. After removing 7412 duplicates, 7497 unique articles remained for screening. Titles and abstracts were reviewed, resulting in the exclusion of 7100 records. Subsequently, 397 full-text articles were assessed for eligibility. The PRISMA flow diagram outlining this process is provided (Figure 1). Ultimately, 14 articles with 15 RCTs were included in the systematic review and meta-analysis.

### Study Characteristics

The characteristics of the 15 included RCTs are presented below (Table S2 in Multimedia Appendix 2 [18-25,40-45]). The sample size ranged from 42 to 415 participants, with a total pooled sample size of 1901 across all studies. The meta-analysis incorporated studies conducted with clinical (n=1), subclinical (n=7), and nonclinical (n=8) populations. Overall, 12 studies used retrieval-based CAs, 3 used generative CAs, and 1 study used both retrieval-based and generative CAs. Regarding interaction modes, 13 studies used text-based CAs, and 3 used multimodal CAs.

### Risk of Bias in Included Studies

**Figure 2:**
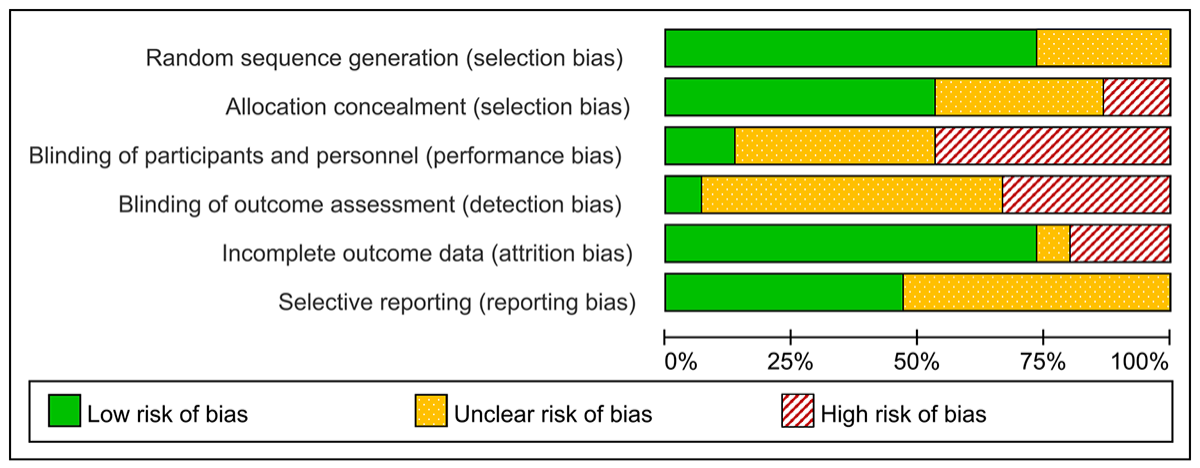
Review authors’ judgements about each risk of bias item.

The overall quality of the included studies was suboptimal. Only one study [41] satisfied all 6 quality criteria. In total, 3 studies met 4 criteria, while 5 studies met 3, and 6 studies met fewer than 3 criteria. Notably, a significant number of studies lacked sufficient information to assess certain criteria: 6 out of 15 studies did not report on the blinding of participants or personnel, 9 did not mention the blinding of outcome assessors, and 8 studies were not registered and lacked information on selective reporting. A summary of the risk of bias for each included randomized controlled trials examining the effect of AI-driven conversational agents in improving mental health among young people is displayed below (Figure 2).

### Depression

#### Overall Effect

**Figure 3:**
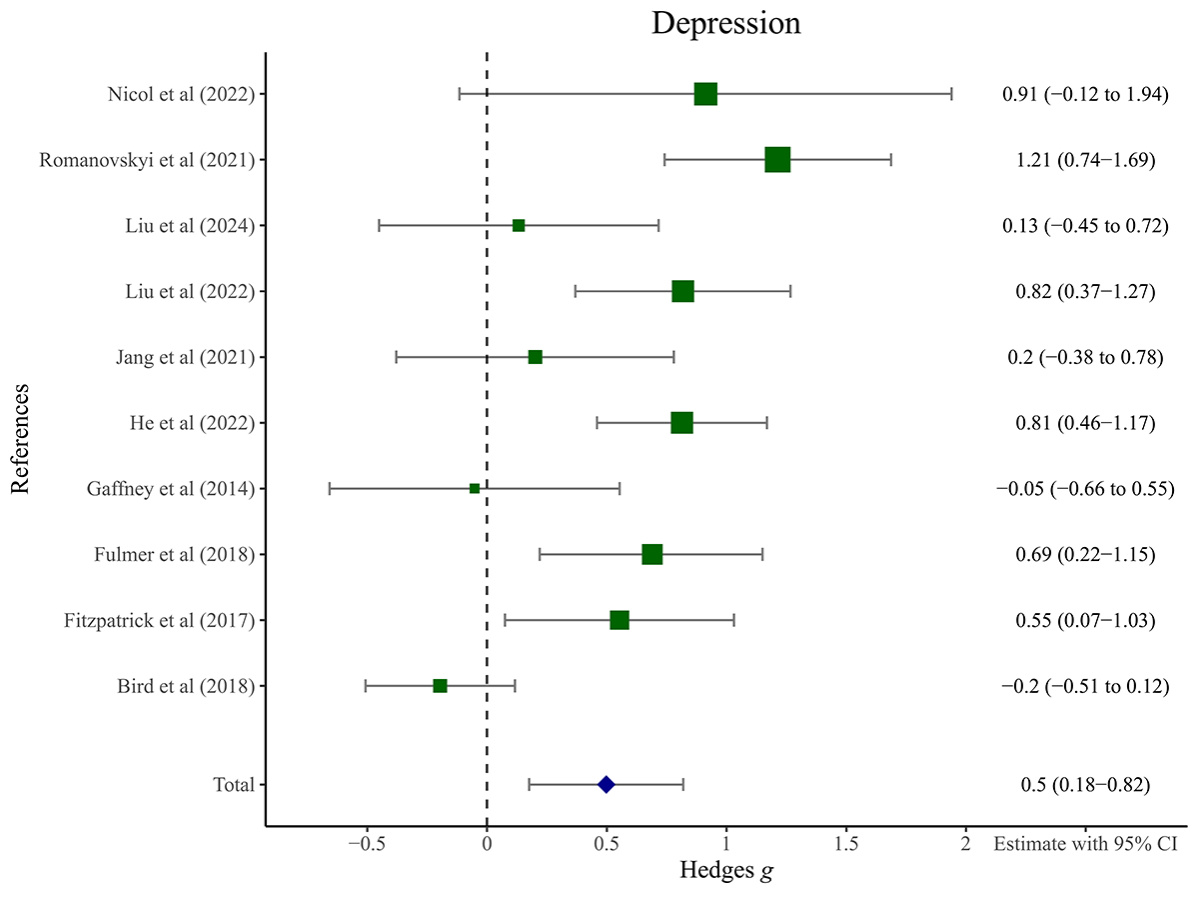
Forest plot for the short-term effects of artificial intelligence–driven conversational agents on depressive symptoms among young people. Hedges g scores (mean and 95% CI) are given (positive values indicate better performance of artificial intelligence–driven conversational agents vs control conditions) [18,19,21,22,24,25,41,42,43,45].

AI-driven CAs demonstrated a medium effect on depression symptoms at posttest, with a Hedges *g* of 0.50 (95% CI 0.18-0.82; N=10; *z*=3.03; *P*<.01). The forest plot was presented in Figure 3 [18,19,21,22,24,25,40-42,44]. Significant heterogeneity was observed among the studies (*Q*_9_=39.64; *P*<.001; *I*²=89.7%). One study [40] was identified as an outlier with 95% CI outside the 95% CI of the pooled studies. After this study was removed, sensitivity analyses indicated that the ES increased to medium-to-large (Hedges *g*=0.61; 95% CI 0.35-0.86; N=9; *z*=4.61; *P*<.001) and the heterogeneity reduced but remained significant (*Q*_8_=17.76; *P*<.05; *I*²=54.9%). This outlier study was excluded from further analyses. Complementary sensitivity analyses confirmed that no single study significantly influenced the results.

#### Publication Bias

Duval and Tweedie’s [37] trim-and-fill method found no evidence of publication bias (Figure S1 in Multimedia Appendix 2). Similarly, the Egger test revealed no significant bias (*b*₀=–2.29, SE=2.04; *t*_7_=1.13; 1-tailed *P*=.15).

#### Moderators

Results of subgroup analyses were presented in Table 2. Hedges *g* scores (mean and 95% CI) are given (positive values indicate better performance of AI-driven conversational agents vs control conditions), along with the number of studies (N) included. *P* value represents the significance of the Cochran *Q* test within subgroups (*Q_w_*) and between subgroups (*Q_b_*). Results revealed that sample type significantly moderated the ESs at posttest (*Q_b_*=8.46, *P*<.05). Only studies conducted in subclinical samples exhibited significant ESs (Hedges *g*=0.74, 95% CI 0.50-0.98), and the ESs were larger than those conducted in nonclinical samples (Hedges *g*=0.04, 95% CI –0.38 to 0.46). In addition, heterogeneities within subgroups were reduced to nonsignificance (*P*>.10). Results of meta-regression analyses were presented in Table 3. *β* coefficients represent the estimated change in the effect size (Hedges *g*) associated with a one-unit increase in the predictor variable. *P* value represents the significance of the *z* test. Results indicated that none of the variables (ie, mean age, publication year, sex, and quality criteria) were significant moderators (*P*>.05).

**Table 2:** Subgroup analyses of short-term efficacy of artificial intelligence–driven conversational agents for depression symptoms among young people.

Moderators		Studies, n	Hedges *g* (95% CI)	*Q*_*w*_	*P*	*Q*_*b*_		*P*	
**Interaction mode**							1.71		.19
	Text-based	7	0.52 (0.17 to 0.86)	15.57	.02				
	Multimodal	2	0.82 (0.54 to 1.09)	0	.99				
**Sample type**							8.46		.02
	Clinical sample	1	0.91 (–0.11 to 1.94)	—^a^	—				
	Nonclinical sample	2	0.04 (–0.38 to 0.46)	0.18	.67				
	Subclinical sample	6	0.74 (0.50 to 0.98)	8.07	.15				
**Control** **g** **roup**							0.61		.74
	Active control	5	0.62 (0.21 to 1.04)	14.77	.005				
	Information only	3	0.52 (0.23 to 0.81)	1.69	.43				
	Waitlist or assessment only	1	0.91 (–0.11 to 1.94)	—	—				
**Delivery platform**							0.06		.97
	Instant messenger platform	5	0.59 (0.15 to 1.03)	11.15	.03				
	Stand-alone application	4	0.62 (0.29 to 0.96)	6.52	.09				
**Intervention length**							1.19		.28
	0-4 weeks	7	0.55 (0.23 to 0.86)	16.79	.01				
	> 4 weeks	2	0.83 (0.42 to 1.24)	0.03	.87				

^a^Not applicable.

**Table 3:** Meta-regression of short-term efficacy of artificial intelligence–driven conversational agents for depression symptoms among young people.

Moderators	Young people, n	Β	SE	*z*	*P*
Mean age	9	–0.06	0.06	–0.98	.33
Year	9	0.05	0.05	0.99	.32
%—total Female	9	–0.01	0.01	–0.69	.49
Quality criteria	9	0.08	0.08	1.02	.31

### Generalized Anxiety

#### Overall Effect

The results showed that AI-driven CAs had a nonsignificant impact on generalized anxiety symptoms compared to control conditions (Hedges *g*=0.42, 95% CI –0.04 to 0.87; N=10; *z*=1.78; *P*=.08; Figure S2 in Multimedia Appendix 2). Large and significant heterogeneity was observed (*Q*_9_=71.45; *P*<.001; *I*²=87.4%). One study [22] was identified as an outlier with 95% CI outside the 95% CI of the pooled studies. After this study was removed, the ES reduced and remained nonsignificant (Hedges *g*=0.17; 95% CI –0.07 to 0.42; N=9; *z*=1.38; *P*=.17) and the heterogeneity remained significant (*Q*_5_=16.41; *P*<.05; *I*²=51.3%). This outlier study was excluded from further analyses. Complementary sensitivity analyses confirmed the robustness of the findings.

#### Publication Bias

Duval and Tweedie’s trim-and-fill analysis indicated that 2 studies were missing on the left side of the mean ES. After imputing the missing studies under a random-effects model, the adjusted ES remained nonsignificant (*g*=0.06, 95% CI –0.21 to 0.32; *Q*_11_=27.20). However, Egger test failed to detect significant publication bias (*b*₀=1.97, SE=1.68; *t*_7_=1.18; 1-tailed *P*=.14).

#### Moderators

Given that the adjusted ES was nonsignificant (*P*>.05), moderator analyses were not conducted.

### Stress

#### Overall Effect

AI-driven CAs had a nonsignificant impact on stress at posttest compared to control groups (Hedges *g*=0.002, 95% CI –0.19 to 0.20; N=4; *z*=0.02; *P*=.98; Figure S3 in Multimedia Appendix 2). No significant heterogeneity was detected among the studies (*Q*_3_=2.39; *P*=.50; *I*²=0.0%). Sensitivity analyses showed that the results were not driven by any single study, and no outliers were identified.

#### Publication Bias

Neither Duval and Tweedie’s trim-and-fill method nor Egger test found any evidence of publication bias (*b*₀=–0.68, SE=1.75; *t*_2_=0.39; 1-tailed *P*=.37; Figure S1 in Multimedia Appendix 2).

#### Moderators

Since the overall ES and heterogeneity were not significant (*P*>.05), moderator analyses were not performed.

### Positive Affect

#### Overall Effect

The effect of AI-driven CAs on positive affect at posttest was nonsignificant (*g*=0.01, 95% CI –0.24 to 0.27; N=7; *z*=0.11; *P*=0.92; Figure S4 in Multimedia Appendix 2). There was significant heterogeneity among the studies (*Q*_6_=16.28; *P*=.01; *I*²=63.1%). Sensitivity analyses confirmed that the results were not driven by any individual study, and no outliers were detected.

#### Publication Bias

Duval and Tweedie’s trim-and-fill method and Egger test (*b*₀=2.12, SE=2.21; *t*_5_=0.96; 1-tailed *P*=.19; Figure S1 in Multimedia Appendix 2) both indicated no publication bias.

#### Moderators

Given the nonsignificant ES (*P*>.05), no moderator analyses were conducted.

### Negative Affect

#### Overall Effect

Similar to the effect on positive affect, AI-driven CAs demonstrated a nonsignificant effect on negative affect compared to control groups at posttest (*g*=0.36, 95% CI –0.04 to 0.76; N=7; *z*=1.77; *P*=.08; Figure S5 in Multimedia Appendix 2). Heterogeneity was large and significant among the studies (*Q*_6_=38.11; *P*<.001; *I*²=84.3%). One study [22] was identified as an outlier with a 95% CI outside the 95% CI of the pooled studies. After this study was removed, the ES reduced and remained nonsignificant (Hedges *g*=0.11, 95% CI –0.06 to 0.28; N=10; *z*=4.95; *P*<.001) and the heterogeneity reduced to nonsignificance (*Q*_5_=5.64; *P*=.34; *I*²=11.3%). This outlier study was excluded from further analyses. Complementary sensitivity analyses confirmed that no study disproportionately influenced the results.

#### Publication Bias

Duval and Tweedie’s trim-and-fill analysis indicated that one study was missing on the left side of the mean ES. After imputing the missing study under a random-effects model, the adjusted ES remained nonsignificant (Hedges *g*=0.07, 95% CI –0.13 to 0.27; *Q*_6_=8.96; Figure S1 in Multimedia Appendix 2). However, Egger test suggested no evidence of publication bias (*b*₀=1.53, SE=1.40; *t*_5_=1.09; 1-tailed *P*=.17).

#### Moderators

Given the nonsignificant overall ES (*P*>.05), no moderator analyses were performed.

### Mental Well-Being

#### Overall Effect

The effect of AI-driven CAs on mental well-being at posttest was nonsignificant (*g*=0.04, 95% CI –0.21 to 0.29; N=4; *z*=0.31; *P*=.76; Figure S6 in Multimedia Appendix 2). There was significant heterogeneity among the studies (*Q*_3_=4.43; *P*=.22; *I*²=32.3%). Sensitivity analyses confirmed that the results were not driven by any individual study, and no outliers were detected.

#### Publication Bias

Duval and Tweedie’s trim-and-fill method and Egger test (*b*₀=3.46, SE=2.01; *t*_2_=1.72; 1-tailed *P*=.11; Figure S1 in Multimedia Appendix 2) both indicated no publication bias.

#### Moderators

Given the nonsignificant ES (*P*>.05), no moderator analyses were conducted.

## Discussion

### Principal Findings

This meta-analysis was the first comprehensive evaluation of the effectiveness of AI-driven CAs mental health intervention among young people. Overall, 16 studies with a total of 1974 participants were evaluated. Findings underscored the potential of AI-driven CAs to significantly alleviate depressive symptoms, particularly in subclinical populations. However, their effects on other mental health outcomes, such as anxiety, stress, and negative affect, were less robust, revealing important insights into both the promise and limitations of AI-driven interventions in this demographic.

### Principal Results and Comparison With Previous Work

The results of this meta-analysis revealed that AI-driven CAs demonstrated a moderate-to-large intervention effect on depressive symptoms. This finding aligns with previous research that has demonstrated the efficacy of AI-driven CAs in reducing depression among all age groups [28], which also revealed that AI-driven CAs had a moderate-to-large effect on depression. This suggests that AI-driven CAs, especially when enhanced with NLP and machine learning, can be particularly effective in mitigating depressive symptoms. To note, these results are more favorable compared with earlier meta-analyses that included non-NLP systems, which reported smaller ESs (Hedges *g* ranging from 0.26 to 0.29) for depression [39,46]. One possible explanation is that as AI-driven CAs can offer greater flexibility and adaptability in delivering therapeutic interventions [47,48], thus they are generally more effective in managing depressive symptoms than their non-NLP counterparts. It is also possible that the larger ES found in this study reflects that AI-based CAs are more beneficial to young people, for whom digital interventions may be more acceptable and engaging due to their familiarity with digital platforms [49]. This aligns with a previous review which indicated that younger age was associated with a larger effect of CAs on depressive symptoms [39].

In contrast to the substantial effects observed for depression, the effects of AI-driven CAs on anxiety, stress, positive affect, negative affect, and mental well-being in this age group were all nonsignificant. This aligns with previous meta-analyses that have found AI-based CA interventions to be less effective for anxiety, positive affect, negative affect, and psychological well-being compared to depression [28,50]. The nonsignificant findings for anxiety and stress in this meta-analysis may be explained by the limited inclusion of behavioral strategies, such as exposure therapy, in current AI-driven CAs. As anxiety and stress often require more intensive behavioral interventions [51], future iterations of AI-driven CAs may benefit from integrating these techniques to improve outcomes for anxiety-related symptoms. In addition, the small and nonsignificant effects on outcomes related to well-being may suggest that AI-based CAs were not yet able to enhance well-being in young people. It is possible that as most AI-driven CAs were based on CBT [47], they are less effective in cultivating positive psychological assets.

Subgroup analyses revealed the significant role of clinical versus subclinical type in moderating the efficacy of AI-driven CAs on depression intervention. Specifically, AI-driven CAs were particularly effective in subclinical populations. This finding aligns with a previous meta-analysis [28], suggesting that subclinical populations are more likely to benefit from AI-based CAs. This is consistent with the broader literature on psychological interventions, which has shown that these interventions are often more effective in promoting mental well-being for people with mental or physical health conditions compared to the general population [52]. Subclinical depression is clinically significant not only because it can cause considerable impairment requiring intervention, but also due to the heightened risk of progression to major depressive disorder, which can potentially be prevented with early treatment [53]. The notable intervention effectiveness of AI-driven CAs among young people with subclinical depression provides an important insight that these digital tools may serve as valuable early interventions to help mitigate the risk of developing more severe mental health conditions. In addition, heterogeneities within studies conducted in clinical and subclinical samples were all nonsignificant (*P*>.10), suggesting that the overall heterogeneity observed in the total sample may be attributed to differential effects of AI-driven CAs across clinical, subclinical, and nonclinical populations.

The nonsignificant moderating effects of interaction mode and delivery platform revealed that CA technical features may not influence the effectiveness of AI-driven CAs in reducing depression among young people. It is possible that as young people are familiar with digital platforms, they can interact effectively with AI-driven CAs with different technical features. Considering the diversity of AI-driven CAs in system orientation, topic constraints, and response patterns, they may meet the needs of different types of users, such as vertical AI-driven CAs (eg, Woebot) [19] where domain knowledge is more structured, intervention is more scientific, and compliant. Open content AI-driven CAs (eg, Replika) [20] can attract users’ attention and establish emotional connections through richer and more diverse dialogue content, making them suitable for early intervention in mental health (such as depression emotion recognition). AI-driven CAs with more structured response methods (eg, Manage Your Life Online) [21] can help individuals with poor self-management abilities to train and those users who are prone to contemplation may prefer to engage in open discussions with freely chatting CAs (eg, ChatGPT) [18]. Since AI-driven CAs can be used in diverse ways depending on user input, it remains unclear what participants were actually doing during these interventions, which also suggests that future research should further explore the impact of individual characteristics on the effectiveness of digital therapy. Age and sex also did not moderate posttest effect. This may reflect that AI-driven CAs could be effective for both males and females and young people in different age groups. In addition, intervention length did not moderate the ESs on depression, which may reflect that both short-course and long-course treatments delivered by AI-driven CAs could be effective in alleviating depressive symptoms. Finally, control group type, publication year, and study quality did not moderate posttest ESs, which further support the robustness of the effectiveness of AI-driven CAs for depression among young people.

### Limitations

Despite the promising results, several limitations should be acknowledged. First, the limited number of studies examining the long-term effects of AI-driven CAs prevented a thorough evaluation of the sustainability of treatment outcomes. As digital interventions continue to gain prominence, it is crucial for future studies to include follow-up assessments to better understand the durability of therapeutic effects. Second, the inclusion of only English-language studies may have introduced selection bias, limiting the generalizability of our findings. Third, some analyses had relatively lower statistical power due to the limited number of studies available for certain outcomes (eg, stress and mental well-being, both with N=4). Similarly, the number of studies in some subgroups was limited, which made it difficult to reach robust conclusions in subgroup analyses. Fourth, we included CAs based on various therapeutic orientations, which may lead to considerable heterogeneity in results. Finally, the current review did not address how users engage with AI-driven CAs. The variability in how people use AI-driven CAs introduces challenges in assessing treatment fidelity and determining whether specific features or therapeutic components of CAs drive effectiveness. Future research should explore user interaction themes and engagement patterns to better understand how AI-driven CAs function in practice and to identify key ingredients contributing to their clinical effectiveness.

### Implications

Practically, the findings from this study support the integration of AI-driven CAs as part of mental health interventions targeting the early stages of depression. Their ability to adapt and tailor their interactions based on user inputs may make them more personalized and effective than early forms of digital mental health interventions. In addition, their accessibility makes them valuable tools, particularly for young people facing barriers to traditional therapy. For young people with depressive disorders, AI-driven CAs may be insufficient as a stand-alone treatment. Future research could explore their potential as an adjunct to traditional therapeutic approaches. To increase their adaptability and effectiveness, incorporating other evidence-based therapeutic approaches—such as adding exposure techniques for anxiety [52]—may enable CAs to better serve diverse mental health needs among young people. For instance, in vivo and imaginal exposure tasks have been successfully integrated into smartphone applications for treating anxiety and stress-related disorders [54], suggesting their potential for integration into AI-driven CAs as well.

### Conclusions

With continued advancements in AI technologies, these digital tools have the potential to play a pivotal role in bridging the mental health treatment gap for young people. This meta-analysis provides robust evidence for the effectiveness of AI-driven CAs in reducing depressive symptoms among young people, particularly in subclinical populations. Their effectiveness for anxiety, stress, and outcomes related to well-being is not robust, highlighting the need for further development. Future research should focus on refining the therapeutic capabilities of AI-driven CAs and exploring long-term mental health outcomes.

## Data Availability

Deidentified data generated or analyzed during this study are available from the corresponding author on reasonable request.

## Appendix

Multimedia Appendix 1 PRISMA (Preferred Reporting Items for Systematic Reviews and Meta-Analyses) checklist.

Multimedia Appendix 2 The supplemental content of search strategies, funnel plots, forest plots, and study characteristics.

## Abbreviations

AI: artificial intelligence
CA: conversational agent
CBT: cognitive behavioral therapy
ES: effect size
NLP: natural language processing
PRISMA: Preferred Reporting Items for Systematic Reviews and Meta-Analyses
RCT: randomized controlled trial
WHO: World Health Organization
