# Supplementary material for: Effectiveness of AI-Driven Conversational Agents in Improving Mental Health Among Young People: Systematic Review and Meta-Analysis
Source: J Med Internet Res. 2025 May 14;27:e69639. doi: 10.2196/69639 (PMC12120367; doi:10.2196/69639)
Supplement: Multimedia Appendix 2 [file jmir_v27i1e69639_app2.docx]

**Effectiveness of AI-driven conversational agents in improving mental health among young people: A systematic review and meta-analysis**

Supplementary material

Contents

[Search strategies 2](#_Toc198049167)

[Supplementary Figure S1. Funnel plots of all outcome variables 6](#_Toc198049168)

[Supplementary Figure S2. Forest plot of AI-driven CAs for generalized anxiety 7](#_Toc198049169)

[Supplementary Figure S3. Forest plot of AI-driven CAs for stress 8](#_Toc198049170)

[Supplementary Figure S4. Forest plot of AI-driven CAs for positive affect 9](#_Toc198049171)

[Supplementary Figure S5. Forest plot of AI-driven CAs for negative affect 10](#_Toc198049172)

[Supplementary Figure S6. Forest plot of AI-driven CAs for mental well-being 11](#_Toc198049173)

[Supplementary Table S2. Study characteristics 12](#_Toc198049174)

## Search strategies

***Web of Science***

TI=( ("mental illness" OR "mental disorder*" OR "affective disorder*" OR "psychotic disorder*" OR "post-traumatic stress disorder*" OR "PTSD" OR distress OR "depress*" OR anxiety OR bipolar OR schizophrenia OR psychosis OR "mental health" OR "mental wellness" OR wellbeing OR "well-being" OR "SWB" OR happiness OR happy OR "positive affect*" OR "negative affect*" OR "positive emotion*" OR "negative emotion*" OR mood OR "life satisfaction" OR "healthy relationship*" OR "resilience" OR "self-efficacy") AND ("robot*" OR "social bot*" OR "dialogue system*" OR "conversational agent*" OR "conversational bot*" OR "conversational system*" OR "conversational interface*" OR "chatbot*" OR "chat bot*" OR "chatterbot*" OR "chatter bot*" OR "chat-bot*" OR "smartbot*" OR "smart bot*" OR "smart-bot*" OR "virtual coach*" OR "virtual agent*" OR "embodied agent*" OR "relational agent*" OR "avatar*" OR "virtual character*" OR "animated character*" OR "virtual human*" OR "virtual assistant*" OR "digital assistant*" OR "counseling agent*") ) OR AB=( ("mental illness" OR "mental disorder*" OR "affective disorder*" OR "psychotic disorder*" OR "post-traumatic stress disorder*" OR "PTSD" OR distress OR "depress*" OR anxiety OR bipolar OR schizophrenia OR psychosis OR "mental health" OR "mental wellness" OR wellbeing OR "well-being" OR "SWB" OR happiness OR happy OR "positive affect*" OR "negative affect*" OR "positive emotion*" OR "negative emotion*" OR mood OR "life satisfaction" OR "healthy relationship*" OR "resilience" OR "self-efficacy") AND ("robot*" OR "social bot*" OR "dialogue system*" OR "conversational agent*" OR "conversational bot*" OR "conversational system*" OR "conversational interface*" OR "chatbot*" OR "chat bot*" OR "chatterbot*" OR "chatter bot*" OR "chat-bot*" OR "smartbot*" OR "smart bot*" OR "smart-bot*" OR "virtual coach*" OR "virtual agent*" OR "embodied agent*" OR "relational agent*" OR "avatar*" OR "virtual character*" OR "animated character*" OR "virtual human*" OR "virtual assistant*" OR "digital assistant*" OR "counseling agent*") )

***Pubmed***

((((((((((((((((((((((((((((((((((((((((mental illness[Title/Abstract]) ) OR (mental disorder*[Title/Abstract])) OR (affective disorder[Title/Abstract])) OR (psychotic disorder[Title/Abstract])) OR (post-traumatic stress disorder[Title/Abstract])) OR (PTSD[Title/Abstract])) OR (distress[Title/Abstract])) OR (depress[Title/Abstract])) OR (anxiety[Title/Abstract])) OR (bipolar[Title/Abstract])) OR (schizophrenia[Title/Abstract])) OR (psychosis[Title/Abstract])) OR (mental health[Title/Abstract])) OR (mental wellness[Title/Abstract])) OR (wellbeing[Title/Abstract])) OR (well-being[Title/Abstract])) OR (SWB[Title/Abstract])) OR (happiness[Title/Abstract])) OR (happy[Title/Abstract])) OR (positive affect[Title/Abstract])) OR (negative affect[Title/Abstract])) OR (positive emotion[Title/Abstract])) OR (negative emotion[Title/Abstract])) OR (mood[Title/Abstract])) OR (life satisfaction[Title/Abstract])) OR (healthy relationship[Title/Abstract])) OR (resilience[Title/Abstract])) OR (self-efficacy[Title/Abstract])) AND (((((((((((((((((((((((((robot[Title/Abstract]) OR (social bot[Title/Abstract])) OR (dialogue system[Title/Abstract])) OR (conversational agent[Title/Abstract])) OR (conversational bot[Title/Abstract])) OR (conversational system[Title/Abstract])) OR (conversational interface[Title/Abstract])) OR (chatbot[Title/Abstract])) OR (chat bot[Title/Abstract])) OR (chatterbot[Title/Abstract])) OR (chatter bot[Title/Abstract])) OR (chat-bot[Title/Abstract])) OR (smartbot[Title/Abstract])) OR (smart bot[Title/Abstract])) OR (smart-bot[Title/Abstract])) OR (virtual coach[Title/Abstract])) OR (virtual agent[Title/Abstract])) OR (embodied agent[Title/Abstract])) OR (relational agent[Title/Abstract])) OR (avatar[Title/Abstract])) OR (virtual character[Title/Abstract])) OR (animated character[Title/Abstract])) OR (virtual human[Title/Abstract])) OR (virtual assistant[Title/Abstract])) OR (digital assistant[Title/Abstract])) OR (counseling agent[Title/Abstract])

***Psychinfo***

AB ( ("mental illness" OR "mental disorder*" OR "affective disorder*" OR "psychotic disorder*" OR "post-traumatic stress disorder*" OR "PTSD" OR distress OR "depress*" OR anxiety OR bipolar OR schizophrenia OR psychosis OR "mental health" OR "mental wellness" OR wellbeing OR "well-being" OR "SWB" OR happiness OR happy OR "positive affect*" OR "negative affect*" OR "positive emotion*" OR "negative emotion*" OR mood OR "life satisfaction" OR "healthy relationship*" OR "resilience" OR "self-efficacy") AND ("robot*" OR "social bot*" OR "dialogue system*" OR "conversational agent*" OR "conversational bot*" OR "conversational system*" OR "conversational interface*" OR "chatbot*" OR "chat bot*" OR "chatterbot*" OR "chatter bot*" OR "chat-bot*" OR "smartbot*" OR "smart bot*" OR "smart-bot*" OR "virtual coach*" OR "virtual agent*" OR "embodied agent*" OR "relational agent*" OR "avatar*" OR "virtual character*" OR "animated character*" OR "virtual human*" OR "virtual assistant*" OR "digital assistant*" OR "counseling agent*" ) ) OR TI ( ("mental illness" OR "mental disorder*" OR "affective disorder*" OR "psychotic disorder*" OR "post-traumatic stress disorder*" OR "PTSD" OR distress OR "depress*" OR anxiety OR bipolar OR schizophrenia OR psychosis OR "mental health" OR "mental wellness" OR wellbeing OR "well-being" OR "SWB" OR happiness OR happy OR "positive affect*" OR "negative affect*" OR "positive emotion*" OR "negative emotion*" OR mood OR "life satisfaction" OR "healthy relationship*" OR "resilience" OR "self-efficacy") AND ("robot*" OR "social bot*" OR "dialogue system*" OR "conversational agent*" OR "conversational bot*" OR "conversational system*" OR "conversational interface*" OR "chatbot*" OR "chat bot*" OR "chatterbot*" OR "chatter bot*" OR "chat-bot*" OR "smartbot*" OR "smart bot*" OR "smart-bot*" OR "virtual coach*" OR "virtual agent*" OR "embodied agent*" OR "relational agent*" OR "avatar*" OR "virtual character*" OR "animated character*" OR "virtual human*" OR "virtual assistant*" OR "digital assistant*" OR "counseling agent*" ) )

***Cochrane library***

#1 (mental illness):ti,ab,kw OR (mental disorder):ti,ab,kw OR (affective disorder):ti,ab,kw OR (psychotic disorder):ti,ab,kw OR (distress):ti,ab,kw

#2 (post-traumatic stress disorder):ti,ab,kw OR (PTSD):ti,ab,kw OR (depress):ti,ab,kw OR (anxiety):ti,ab,kw OR (bipolar):ti,ab,kw

#3 (schizophrenia):ti,ab,kw OR (psychosis):ti,ab,kw OR (mental health):ti,ab,kw OR (mental wellness):ti,ab,kw OR (life satisfaction):ti,ab,kw

#4 (mental wellness):ti,ab,kw OR (wellbeing):ti,ab,kw OR (well-being):ti,ab,kw OR (SWB):ti,ab,kw OR (resilience):ti,ab,kw

#5 (happiness):ti,ab,kw OR (happy):ti,ab,kw OR (positive affect):ti,ab,kw OR (negative affect):ti,ab,kw OR (positive emotion):ti,ab,kw

#6 (negative emotion):ti,ab,kw OR (mood):ti,ab,kw OR (healthy relationship):ti,ab,kw OR (self-efficacy):ti,ab,kw

#7 (robot):ti,ab,kw OR (social bot):ti,ab,kw OR (dialogue system):ti,ab,kw OR (conversational agent):ti,ab,kw OR (conversational bot):ti,ab,kw

#8 (chatbot):ti,ab,kw OR (conversational interface):ti,ab,kw OR (chat bot):ti,ab,kw OR (chatterbot):ti,ab,kw OR (chatter bot):ti,ab,kw

#9 (chat-bot):ti,ab,kw OR (smartbot):ti,ab,kw OR (smart bot):ti,ab,kw OR (smart-bot):ti,ab,kw OR (virtual agent):ti,ab,kw

#10 (virtual coach):ti,ab,kw OR (embodied agent):ti,ab,kw OR (relational agent):ti,ab,kw OR (avatar):ti,ab,kw OR (virtual character):ti,ab,kw

#11 (animated character):ti,ab,kw OR (virtual human):ti,ab,kw OR (virtual assistant):ti,ab,kw OR (digital assistant):ti,ab,kw OR (counseling agent):ti,ab,kw

#12 #1 or #2 or #3 or #4 or #5 or #6

#13 #7 or #8 or #9 or #10 or #11

#14 #12 and #13

***Embase***

#1 ('mental illness' OR 'mental disorder' OR 'affective disorder' OR 'psychotic disorder' OR 'post-traumatic stress disorder' OR 'PTSD' OR 'distress' OR 'depress' OR 'anxiety' OR 'bipolar' OR 'schizophrenia' OR 'psychosis' OR 'mental health' OR 'mental wellness' OR 'wellbeing' OR 'well-being' OR 'SWB' OR 'happiness' OR 'happy' OR 'positive affect' OR 'negative affect' OR 'positive emotion' OR 'negative emotion' OR 'mood' OR 'life satisfaction' OR 'healthy relationship' OR 'resilience' OR 'self-efficacy'):ab,ti

#2('robot' OR 'social bot' OR 'dialogue system' OR 'conversational agent' OR 'conversational bot' OR 'conversational system' OR 'conversational interface' OR 'chatbot' OR 'chat bot' OR 'chatterbot' OR 'chatter bot' OR 'chat-bot' OR 'smartbot' OR 'smart bot' OR 'smart-bot' OR 'virtual coach' OR 'virtual agent' OR 'embodied agent' OR 'relational agent' OR 'avatar' OR 'virtual character' OR 'animated character' OR 'virtual human' OR 'virtual assistant' OR 'digital assistant' OR 'counseling agent'):ab,ti

#3 #1 AND #2

***MEDLINE***

AB ( ("mental illness" OR "mental disorder*" OR "affective disorder*" OR "psychotic disorder*" OR "post-traumatic stress disorder*" OR "PTSD" OR distress OR "depress*" OR anxiety OR bipolar OR schizophrenia OR psychosis OR "mental health" OR "mental wellness" OR wellbeing OR "well-being" OR "SWB" OR happiness OR happy OR "positive affect*" OR "negative affect*" OR "positive emotion*" OR "negative emotion*" OR mood OR "life satisfaction" OR "satisfaction with life" OR "healthy relationship*" OR "resilience" OR "self-efficacy") AND ("robot*" OR "social bot*" OR "dialogue system*" OR "conversational agent*" OR "conversational bot*" OR "conversational system*" OR "conversational interface*" OR "chatbot*" OR "chat bot*" OR "chatterbot*" OR "chatter bot*" OR "chat-bot*" OR "smartbot*" OR "smart bot*" OR "smart-bot*" OR "virtual coach*" OR "virtual agent*" OR "embodied agent*" OR "relational agent*" OR "avatar*" OR "virtual character*" OR "animated character*" OR "virtual human*" OR "virtual assistant*" OR "digital assistant*" OR "counseling agent*" ) ) OR TI ( ("mental illness" OR "mental disorder*" OR "affective disorder*" OR "psychotic disorder*" OR "post-traumatic stress disorder*" OR "PTSD" OR distress OR "depress*" OR anxiety OR bipolar OR schizophrenia OR psychosis OR "mental health" OR "mental wellness" OR wellbeing OR "well-being" OR "SWB" OR happiness OR happy OR "positive affect*" OR "negative affect*" OR "positive emotion*" OR "negative emotion*" OR mood OR "life satisfaction" OR "satisfaction with life" OR "healthy relationship*" OR "resilience" OR "self-efficacy") AND ("robot*" OR "social bot*" OR "dialogue system*" OR "conversational agent*" OR "conversational bot*" OR "conversational system*" OR "conversational interface*" OR "chatbot*" OR "chat bot*" OR "chatterbot*" OR "chatter bot*" OR "chat-bot*" OR "smartbot*" OR "smart bot*" OR "smart-bot*" OR "virtual coach*" OR "virtual agent*" OR "embodied agent*" OR "relational agent*" OR "avatar*" OR "virtual character*" OR "animated character*" OR "virtual human*" OR "virtual assistant*" OR "digital assistant*" OR "counseling agent*" ) )

## Supplementary Figure S1. Funnel plots of all outcome variables


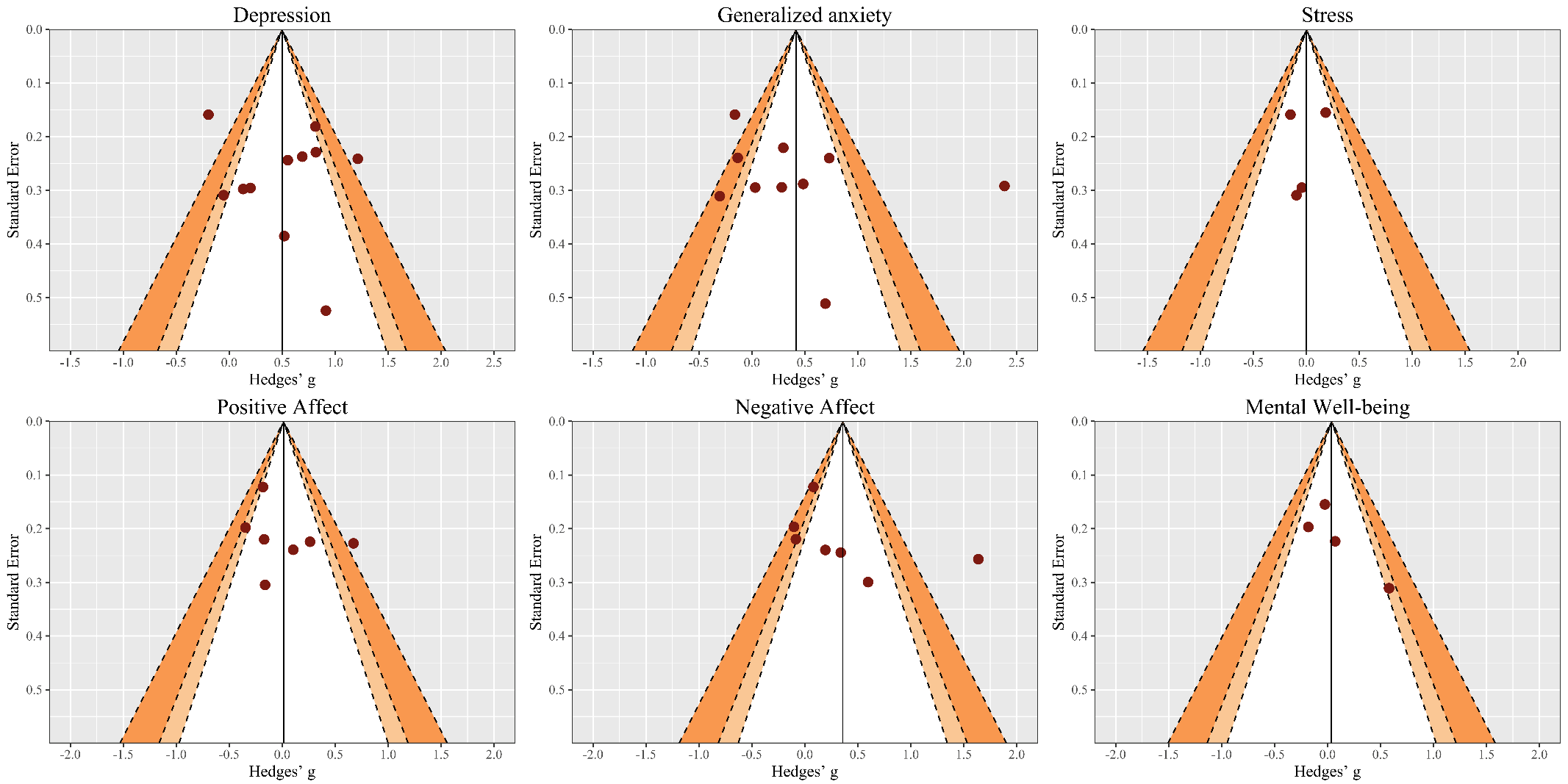


Figure S1. Funnel plots of all outcome variables

*Note.* Each point represents a reference. The areas indicate ranges of two-tailed *p*-values from various studies: white areas correspond to *p* > 0.10, light orange areas to *p* < 0.05, orange areas to *p* < 0.01, and light grey areas outside the main triangle indicate *p* < 0.001.

## Supplementary Figure S2. Forest plot of AI-driven CAs for generalized anxiety


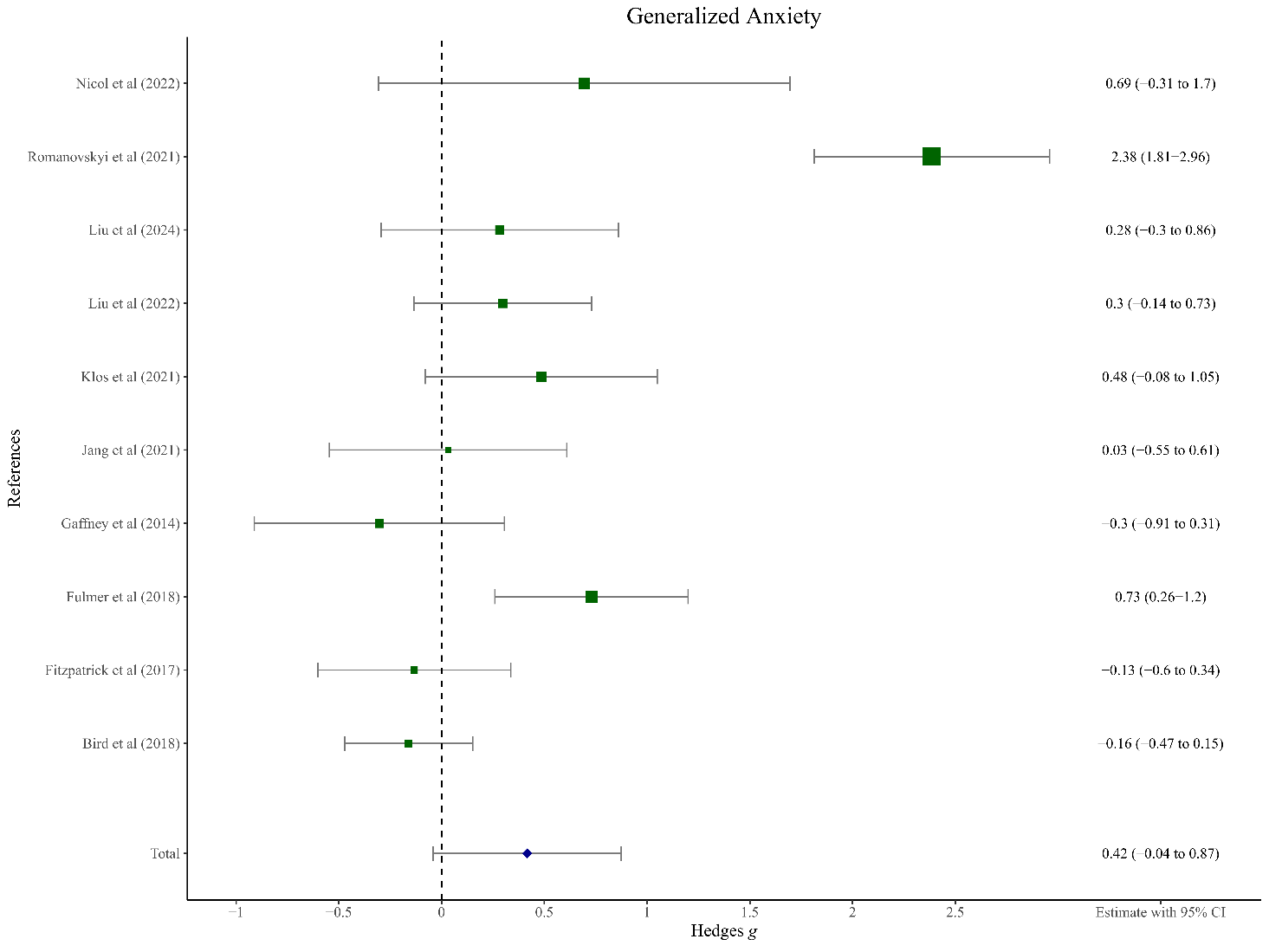


Figure S2. Forest plot of AI-driven CAs for generalized anxiety

*Note.* The green and blue dots represent the total effect size; the size of the dots indicates the size of effect size; the error bars represent the 95% confidence interval.

## Supplementary Figure S3. Forest plot of AI-driven CAs for stress


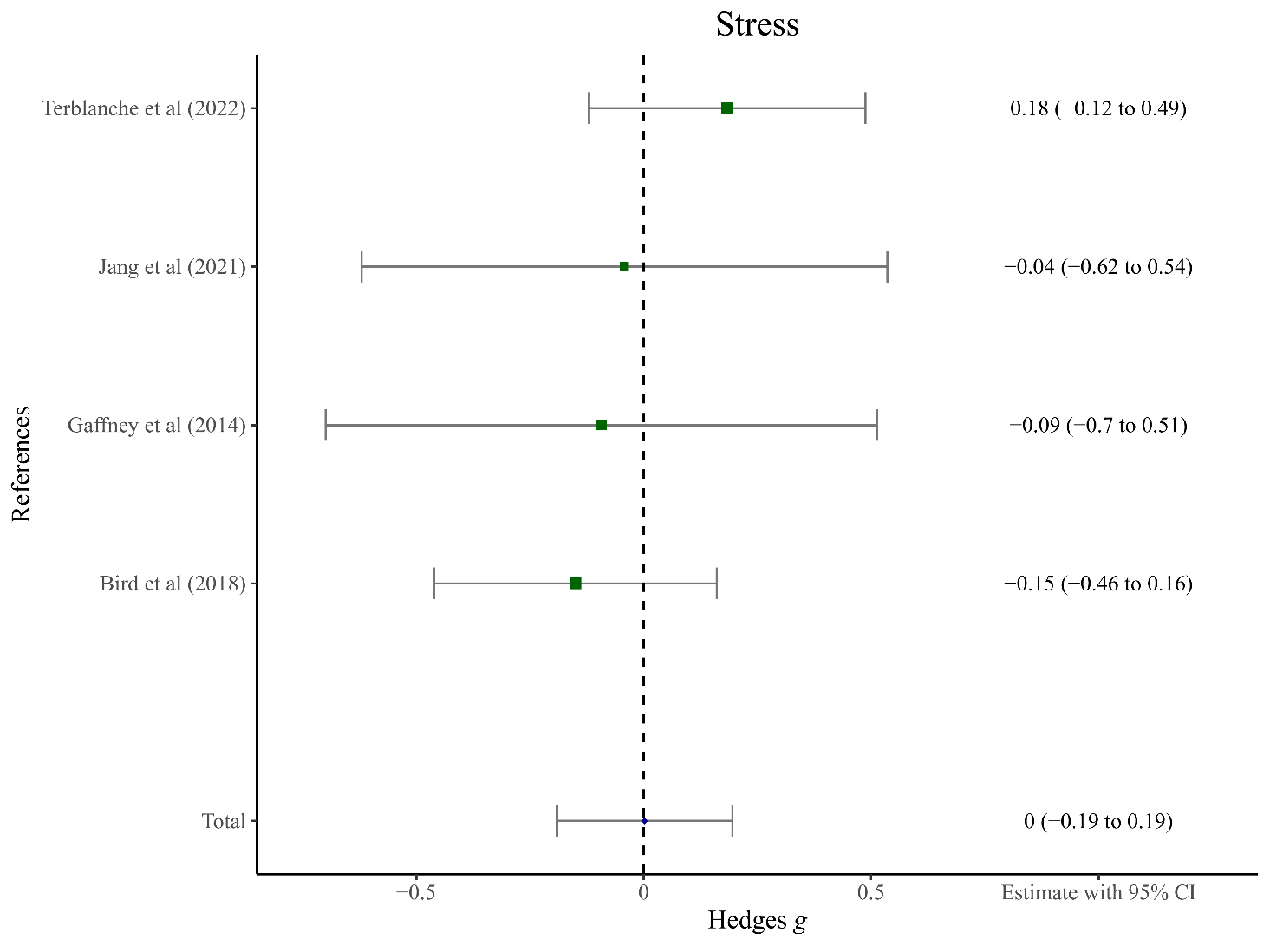


Figure S3. Forest plot of AI-driven CAs for stress

*Note.* The green and blue dots represent the total effect size; the size of the dots indicates the size of effect size; the error bars represent the 95% confidence interval.

## Supplementary Figure S4. Forest plot of AI-driven CAs for positive affect


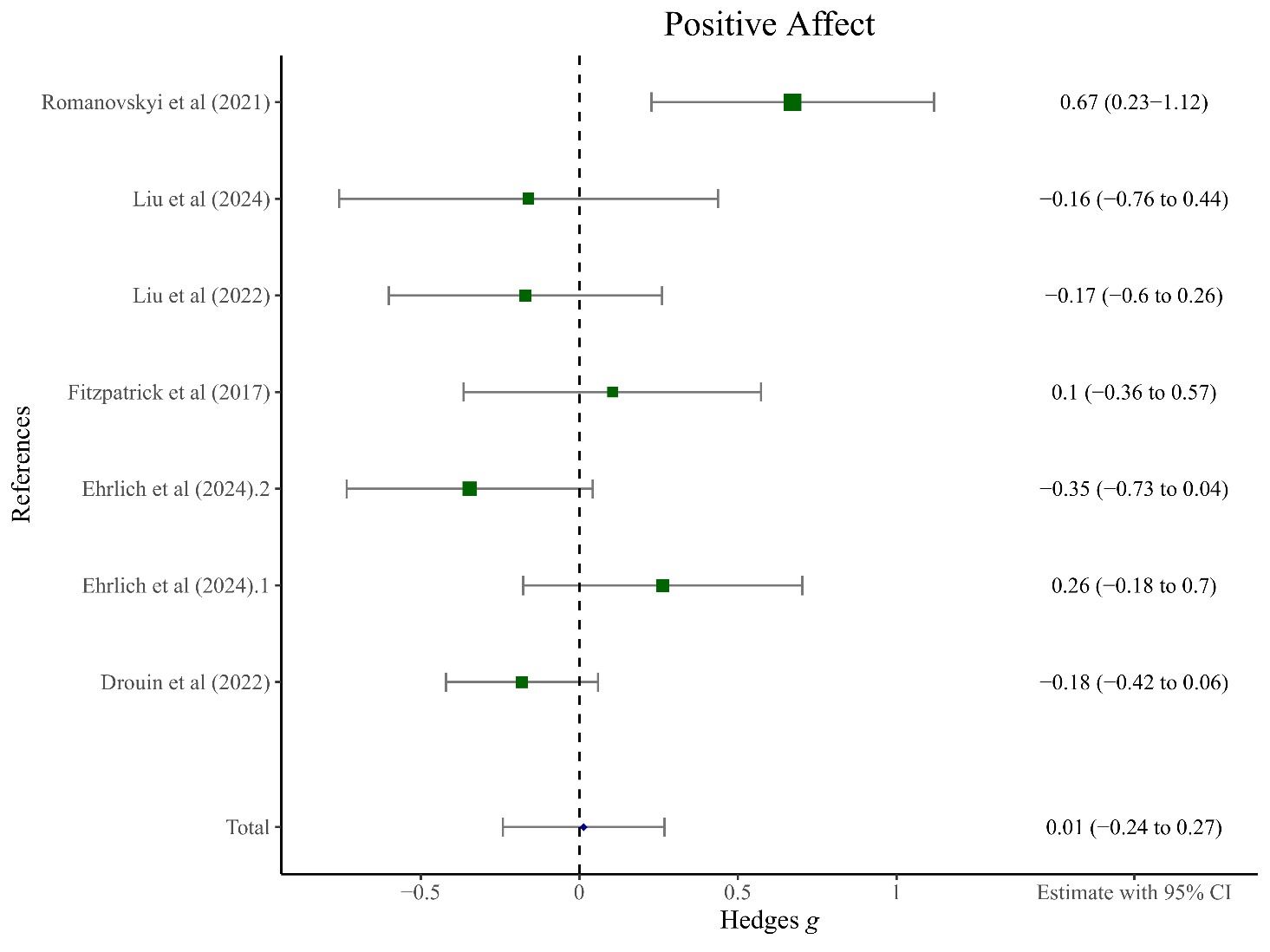


Figure S4. Forest plot of AI-driven CAs for positive affect

*Note.* The green and blue dots represent the total effect size; the size of the dots indicates the size of effect size; the error bars represent the 95% confidence interval.

## Supplementary Figure S5. Forest plot of AI-driven CAs for negative affect


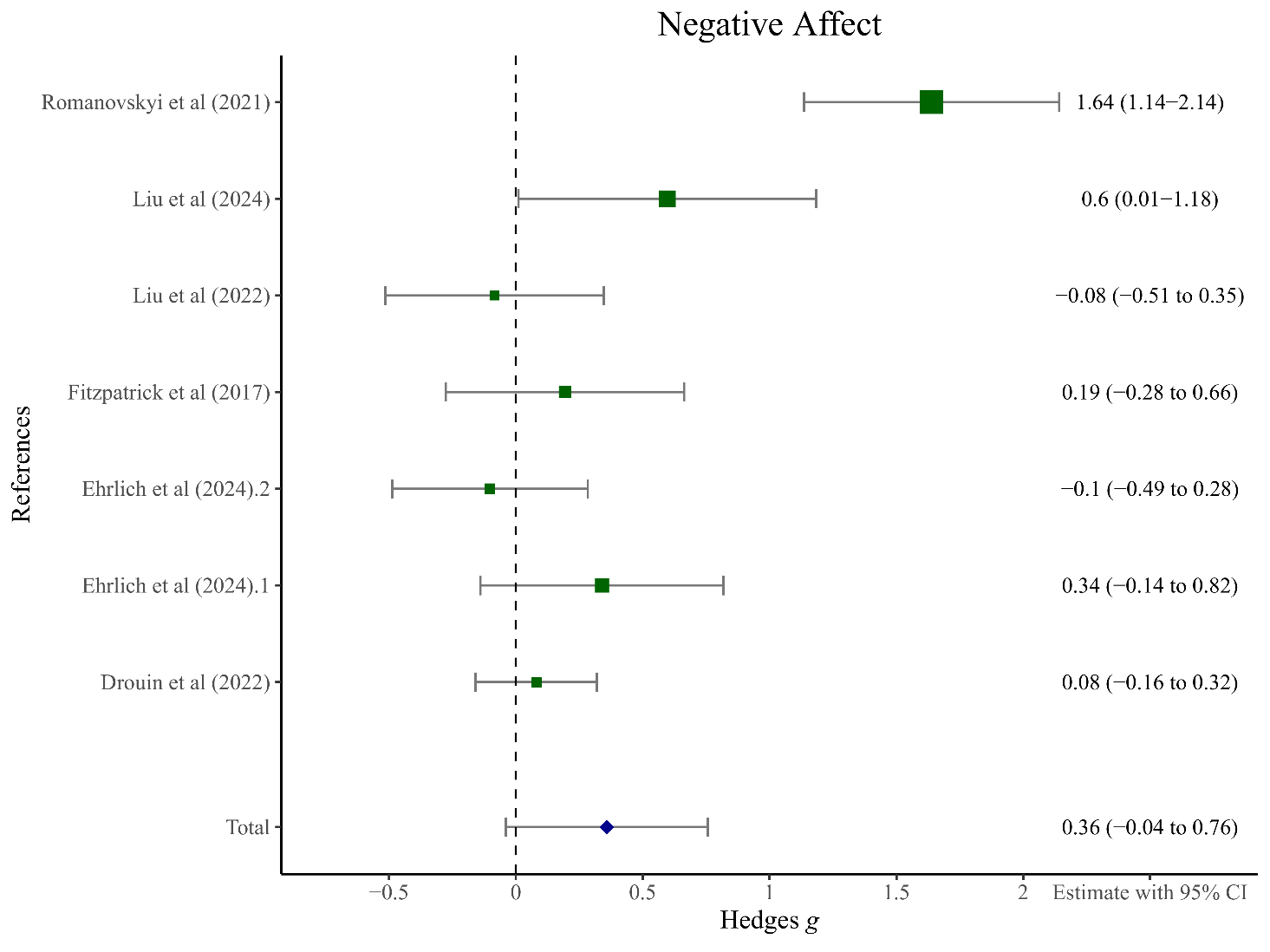


Figure S5. Forest plot of AI-driven CAs for negative affect

*Note.* The green and blue dots represent the total effect size; the size of the dots indicates the size of effect size; the error bars represent the 95% confidence interval.

## Supplementary Figure S6. Forest plot of AI-driven CAs for mental well-being


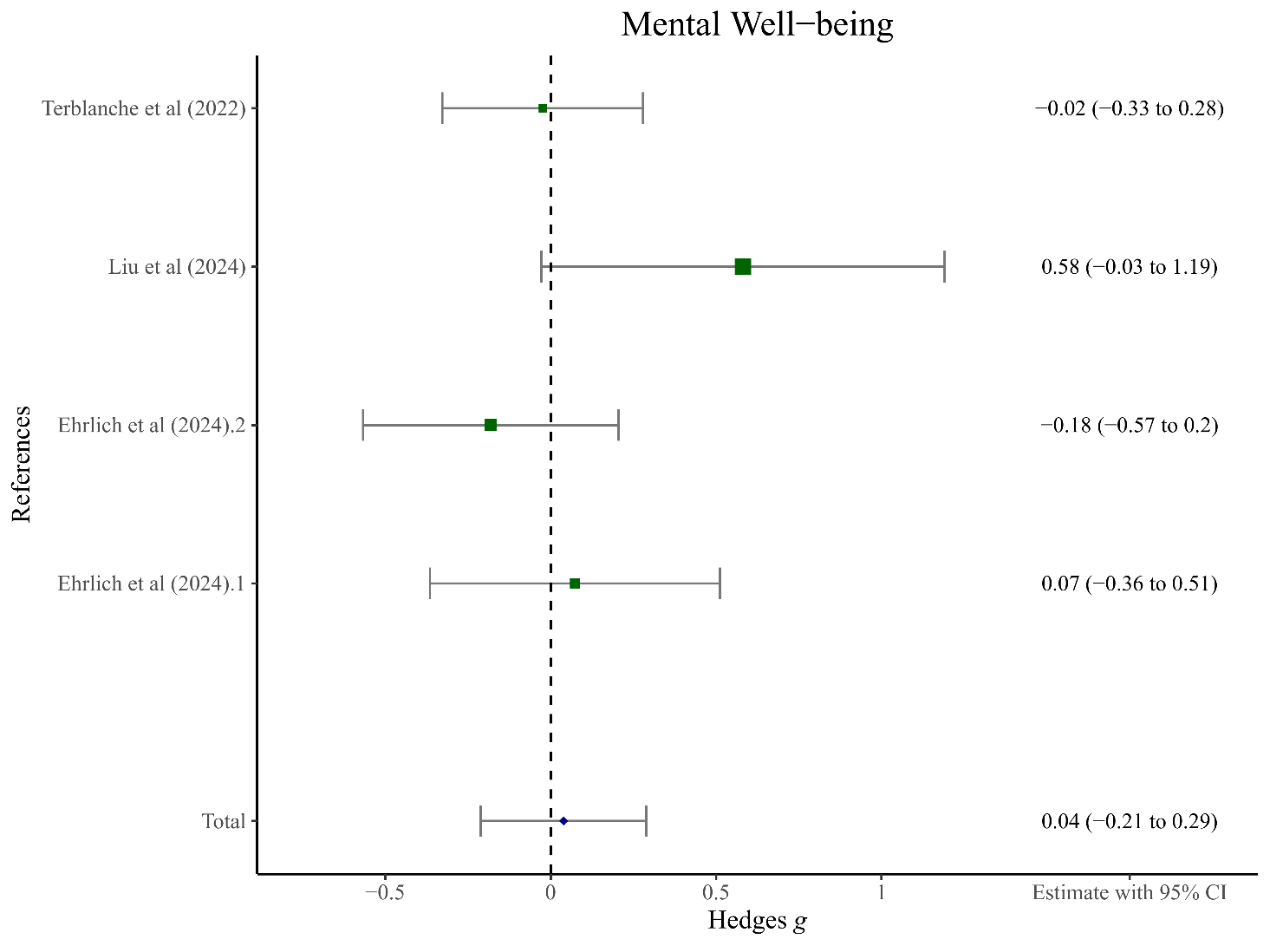


Figure S6. Forest plot of AI-driven CAs for mental well-being

*Note.* The green and blue dots represent the total effect size; the size of the dots indicates the size of effect size; the error bars represent the 95% confidence interval.

## Supplementary Table S2. Study characteristics

| Author (Year) | Sample type | *N* | %—total Female | Mean  age | CA name | Delivery platform | Interaction mode | Therapeutic orientation | Intervention length | | Control | Outcome measures |
| --- | --- | --- | --- | --- | --- | --- | --- | --- | --- | --- | --- | --- |
| Bird et al. (2018) | nonclinical | 171 | 81.6 | 22.08 | MYLO | Instant messenger platform | Text-based | MOL | | 12 min | Active control | DASS-21 |
| Drouin et al. (2022) | nonclinical | 415 | 71.2 | 19.82 | Replika | standalone app | Multimodal | NR | | 20 min | Active control | PANAS |
| Ehrlich et al. (2024).1 | nonclinical | 177 | 52.0 | 20.80 | Mind Tutor | standalone app | Text-based | Integrative approach | | 6 weeks | Waitlist | SWEMWBS, SWLS, PANAS-SF,  GSES |
| Ehrlich et al. (2024).2 | nonclinical | 250 | 56.0 | 20.23 | Mind Tutor | standalone app | Text-based | Integrative approach | | 6 weeks | Waitlist | SWEMWBS, SWLS, PANAS-SF, GSES |
| Fitzpatrick et al. (2017) | subclinical | 70 | 67.0 | 22.20 | Woebot | standalone app | Text-based | CBT | | 2 weeks | Information only | PHQ-9, GAD-7, PANAS |
| Gaffney et al. (2014) | nonclinical | 42 | 78.6 | 21.40 | MYLO | Web-based | Text-based | MOL | | 20 min | Active control | DASS-21 |
| Fulmer et al. (2018) | subclinical | 75 | 69.0 | 22.90 | Tess | Instant messenger platform | Text-based | Integrative | | 2-4 weeks | Information only | GAD-7, PHQ-9 |
| He et al. (2022) | subclinical | 125 | 80.0 | 18.78 | XiaoE | Instant messenger platform | Multimodal | CBT | | 1 weeks | Active control | PHQ-9 |
| Jang et al. (2021) | subclinical | 46 | 57.0 | 24.79 | Todaki | standalone app | Text-based | CBT | | 4 weeks | Information only | QIDS-SR, SAS, PSS |
| Klos et al. (2021) | nonclinical | 73 | 87.0 | NR | Tess | Instant messenger platform | Text-based | Integrative | | 8 weeks | Information only | PHQ-9, GAD-7 |
| Liu et al. (2022) | subclinical | 83 | 55.0 | 23.08 | XiaoNan | Instant messenger platform | Multimodal | CBT | | 16 weeks | Active control | PHQ-9, GAD-7, PANAS |
| Liu et al. (2024) | nonclinical | 48 | 41.8 | 21.14 | ChatGPT-3.5-Turbo API | standalone app | Text-based | PPI | | 2 weeks | Active control | PHQ-9, GAD-7, PANAS, SWLS, PWB |
| Romanovskyi et al. (2021) | subclinical | 82 | 48.8 | 20.85 | Elomia | standalone app | Text-based | Integrative approach | | 4 weeks | Active control | PHQ-9, GAD-7, PANAS |
| Terblanche et al. (2022) | nonclinical | 169 | 56.0 | 22.00 | Vici | Instant messenger platform | Text-based | GROW coaching model | | 6 months | Waitlist | WEMWBS, BRS, PSS-10 |
| Nicol et al. (2022) | clinical | 75 | 88.2 | 14.80 | Woebot | standalone app | Text-based | CBT | | 12 weeks | Waitlist | PHQ-9, GAD-7, MHSES |

*Notes.* CA = conversational agent; MOL = method of levels therapy; CBT = cognitive behavioral therapy; DASS-21 = Depression, Anxiety, and Stress Scales-Short form; NR = not report; PANAS = Positive and Negative Affect Schedule; SWEMWBS = Short Warwick Edinburgh Mental Well-being Scale; SWLS = Satisfaction With Life Scale; PANAS-SF = Positive and Negative Affect Schedule-Short form; GSES = General Self-Efficacy Scale; PHQ-9 = Patient Health Questionnaire-9; GAD-7 = Generalized Anxiety Disorder scale; QIDS-SR = Quick Inventory of Depressive Symptoms-Self-Report; SAS = Self-rating Anxiety Scale; PSS = Perceived Stress Scale; PWB = Scales of Psychological Well-being; HAD-S = Hospital Anxiety and Depression Scale; WEMWBS = Warwick-Edinburgh Mental Wellbeing Scale; BRS = Brief Resilience Scale; PSS-10 = Perceived Stress Scale-10; MHSES = Mental Health Self-Efficacy Scale.
